# Aberrant Expression of MicroRNA-15a and MicroRNA-16 Synergistically Associates with Tumor Progression and Prognosis in Patients with Colorectal Cancer

**DOI:** 10.1155/2014/364549

**Published:** 2014-11-04

**Authors:** Guangfa Xiao, Huihuan Tang, Wei Wei, Jian Li, Liandong Ji, Jie Ge

**Affiliations:** Department of General Surgery, Xiangya Hospital, Central South University, No. 87 Xiangya Road, Changsha, Hunan 410008, China

## Abstract

The aim of this study was to reveal the associations of microRNA miR-15a and miR-16 dysregulation with clinicopathological characteristics and prognosis in patients with colorectal cancer. As a result, we found that miR-15a and miR-16 expression, detected by quantitative real time-PCR, were both significantly downregulated in colorectal cancer tissues compared with adjacent colorectal mucosa (both *P* < 0.001). Particularly, the expression levels of miR-15a in colorectal cancer tissues were positively correlated with those of miR-16 significantly (Spearman correlation coefficient *r* = 0.652, *P* < 0.001). In addition, miR-15a and/or miR-16 downregulation were all significantly associated with advanced TNM stage (all *P* < 0.05), poorly histological grade (all *P* < 0.05), and positive lymph node metastasis (all *P* < 0.05). Moreover, the survival analysis identified miR-15a expression, miR-16 expression, and miR-15a/miR-16 combination as independent predictors of both unfavorable overall survival and disease-free survival. Interestingly, the prognostic value of miR-15a/miR-16 combination was more significant than miR-15a or miR-16 expression alone. Collectively, the aberrant expression of miR-15a and miR-16 could be used to stratify patients with aggressive tumor progression of colorectal cancer. The combined pattern of miR-15a and miR-16 downregulation has a significant value for distinguishing patients with a worse prognosis of colorectal cancer after surgery.

## 1. Introduction

Colorectal cancer, a common type of gastrointestinal malignancies, represents the third most frequent cancer worldwide and the second leading cause of cancer-related death in the developed countries [1]. Although it has traditionally been recognized to be more common in Western populations, the incidence of colorectal cancer in the most recent years has been continuously and rapidly rising in several Asian countries, such as China, Korea, Japan, and Singapore [2]. As a clinically heterogeneous-multifocal disease, colorectal cancer has a multistep process in the carcinogenesis, progression, and prognosis. Although the 5-year overall survival rate of colorectal cancer has been reported to be as high as 71.3%, the survival rate in patients with recurrence is only 40% [3]. Nearly 50% of colorectal cancer patients die from distant metastases within 6 years of the initial diagnosis. Liver is the predominant and often the only organ for distant metastasis [4]. Several clinicopathological characteristics, such as the tumor-node-metastasis (TNM) staging system, which is based on features of primary tumor, presence and extent of lymph node involvement, and distant metastasis, have been used to assess prognosis of patients with colorectal cancer [5]. However, the patients' clinical outcome still varies greatly among patients within the same stage. Thus, it is extremely necessary to clarify the molecular mechanisms underlying the carcinogenesis of colorectal cancer in order to explore novel markers for predicting the progression and prognosis of this malignancy and guide clinicians to select appropriate treatment.

MicroRNAs (miRNAs), a group of highly conserved and small noncoding RNAs with 18–25 nucleotides, function as posttranscriptional regulators silencing the expression levels of their mRNA targets by interacting with complementary sites in the 3′ untranslated region (3′UTR) [6]. More than 60% of human protein encoding genes are under selective pressure to maintain pairing with miRNAs, implying that miRNAs may play important roles in governing diverse physiological and pathological processes, including cell proliferation, differentiation, apoptosis, and carcinogenesis [7]. Particularly in human cancers, miRNAs can act as either tumor suppressor genes or oncogenes according to the functions of their mRNA targets. Different from most currently available biomarkers, miRNA expression appears to be cell type- and disease-specific patterns and can be used for the classification of certain cancer histotypes [8]. An increasing number of studies have detected various miRNAs with aberrant expression in colorectal cancer, which can accurately differentiate malignant tissues from benign colorectal mucosa. Ak et al. [9] detected the expression profiles of 38 different miRNAs associated with colorectal cancer using miRNA polymerase chain reaction arrays in tumors and surgical margin tissue samples obtained from 40 sporadic early-onset Turkish colorectal cancer patients and found that miR-106a and miR-125b were associated with the formation and invasion of colorectal tumors; Wang et al. [10] built a serum miRNA expression profile signature and tested its specificity and sensitivity as a biomarker in the diagnosis of colorectal cancer.

The miR-15a and miR-16 (miR-15a/16) cluster is localized at chromosome 13q14.3, a genomic region which is frequently deleted in the majority of chronic lymphocytic leukemia (CLL), and in a subset of mantle cell lymphoma [11, 12]. MiRNAs encoded by the miR-15a/16 cluster are frequently deleted or downregulated in many cancer cell lines and various tumor tissues, including CLL, lymphoma, breast cancer, non-small cell lung cancer, hepatocellular carcinoma, gastric cancer, and colorectal cancer, implying their tumor suppressive roles in tumor progression of these malignancies [11–16]. Particularly, Ma and colleagues showed that overexpression of miR-16 could inhibit the proliferation and induce apoptosis of colorectal cancer cells through the intrinsic apoptosis pathway [17]; Qian and colleagues identified low miR-16 expression as an independent factor predicting a poor prognosis for patients with colorectal cancer [18]; Dai and colleagues found that miR-15a/16-1 could be applied in colon cancer treatment in vivo and resulted in effective colon tumor xenografts growth arrest and angiogenesis decrease [19]; Shi et al. also reported that p53-induced miR-15a/16-1 and AP4 could form a double-negative feedback loop to regulate epithelial-mesenchymal transition and metastasis in colorectal cancer [20]. However, the clinical significance of miR-15a/16 cluster in colorectal cancer has not been fully elucidated. Thus, the present study aimed to reveal the associations of miR-15a and miR-16 dysregulation with clinicopathological characteristics and prognosis in patients with colorectal cancer.

## 2. Materials and Methods

### 2.1. Study Population

The protocol of the present study was approved by the Institutional Research Ethics Board of Xiangya Hospital of Central South University. A written informed consent was obtained from each subject enrolled in the present study.

One hundred and twenty-six patients with colorectal cancer (76 men and 50 women), who underwent radical resection at the Department of General Surgery, Xiangya Hospital, Central South University, between February 2001 and January 2003, were enrolled in the present study. The median age of patients at the time of admission was 66 years, range 22–82 years. None of the patients had received either radiotherapy or chemotherapy preoperatively. All diagnoses were confirmed histopathologically, and the paraffin blocks of all primary tumor specimens were available for analysis. Only patients with sporadic colorectal cancer were selected for our analysis, and patients with a positive medical history for hereditary non-polyposis colorectal cancer or familial adenomatous polyposis were excluded at the time of patients' collection. The sixth edition of American Joint Committee on Cancer (AJCC) TNM staging system was used for tumor staging. The detail information on the clinicopathologic characteristics of all 126 patients with colorectal cancer in this study was shown in Table 1. In addition, 126 adjacent colorectal mucosas were surgically excised from the same patients and confirmed as normal by histology.

All 126 patients with colorectal cancer were given a follow-up exam (direct evaluation or phone interview) ranging from 1 to 10 years (median 6.18 years). Patients who died from diseases other than colorectal cancer or from unexpected events were excluded from the case collection in this study. For the analysis of survival and follow-up, the date of curative surgery was used to represent the beginning of the follow-up period. Disease-free survival (DFS) was defined as the length of time from curative surgery to the first tumor recurrence and distant metastasis. Overall survival (OS) was defined as the time from curative surgery to death from any cause.

### 2.2. Quantitative Real Time-PCR (qRT-PCR)

Expression levels of miR-15a and miR-16 in 126 pairs of fresh tumor samples matched with adjacent colorectal mucosa obtained from 126 patients with colorectal cancer were detected by qRT-PCR. Total RNA was isolated with Trizol (Invitrogen, Carlsbad, CA) according to the manufacturer's instructions. First-strand cDNA synthesis was carried out using TaqMan MicroRNA Reverse Transcription kit and RT primers specific to the various miRNAs (Applied Biosystems, Foster City, CA, USA). Real-time PCR was carried out with TaqMan Universal Master Mix II and TaqMan MicroRNA Assay Mix on an ABI PRISM 7500 (Applied Biosystems). U6 RNA was used as an internal control to correct variations in the experiment: the RT-primer for miR-15a: 5′-GTC GTA TCC AGT GCA GGG TCC GAG GTA TTC GCA CTG GAT ACG ACC ACA AAC-3′; the PCR primer for miR-15a: forward 5′-GCG GCT AGC AGC ACA TAA TGG-3′, reverse 5′-GTG CAG GGT CCG AGG T-3′; the RT-primer for miR-16: 5′-GTC GTA TCC AGT GCA GGG TCC GAG GTA TTC GCA CTG GAT ACG ACC ACA AGT-3′; the PCR primer for miR-16: forward 5′-GCG GCA ACC CGT AGA TCC GAA-3′, reverse 5′-GTG CAG GGT CCG AGG T-3′; the PCR primer for U6: forward 5′-CTC GCT TCG GCA GCA CA-3′, reverse 5′-AAC GCT TCA CGA ATT TGC GT-3′. Expression of miRNA was defined based on the threshold cycle (Ct). The 2^−ΔΔCT^ method was used to calculate the relative expression levels of miR-15a or miR-16. Each sample was assessed in triplicate for each miRNA.

### 2.3. Statistical Analysis

Statistical analyses were performed using the SPSS software package (version 13.0; SPSS Inc, IL, USA). miR-15a or miR-16 expression in colorectal cancer tissues and adjacent normal mucosa was compared by paired two tailed *t*-tests. The *χ* ^2^-test or Fisher's exact test was employed to evaluate the association between miR-15a/miR-16 expression and clinicopathological characteristics. The correlation between miR-15a and miR-16 expression was determined by Spearman's rank correlation analysis. The survival analysis was estimated by the Kaplan-Meier method and was compared by using the log-rank test. Multivariate analysis was performed using the Cox proportional hazard model. Differences were considered significant if *P* < 0.05.

## 3. Results

### 3.1. Downregulation of miR-15a and miR-16 in Human Colorectal Cancer Tissues

**Figure 1:**
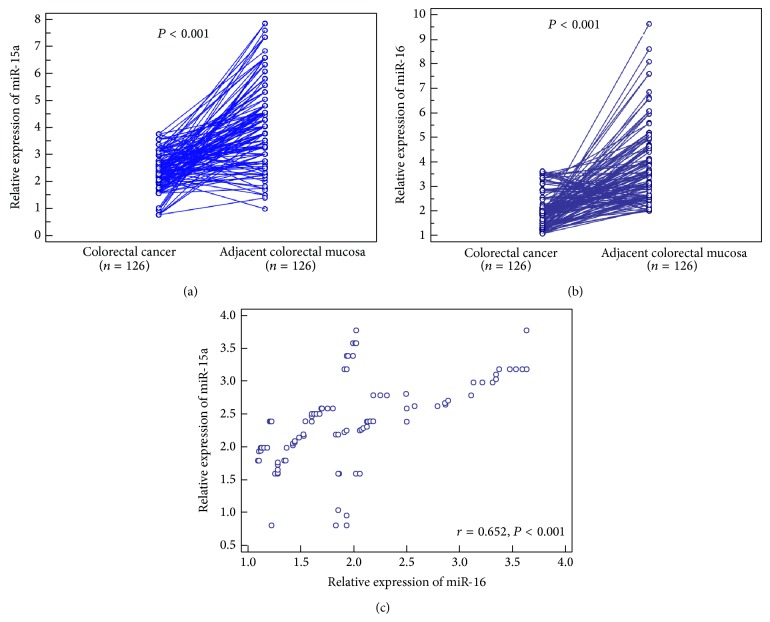
Relative expression levels of miR-15a (a) and miR-16 (b) in colorectal cancer tissues and adjacent colorectal mucosa. The expression levels of miR-15a in colorectal cancer tissues were positively correlated with those of miR-16 significantly (Spearman correlation coefficient *r* = 0.652, *P* < 0.001, (c)).

Compared with adjacent colorectal mucosa, miR-15a (cancer versus normal: 2.38 ± 0.69 versus 4.20 ± 1.55, *P* < 0.001, Figure 1(a)) and miR-16 (cancer versus normal: 2.02 ± 0.71 versus 3.77 ± 1.46, *P* < 0.001, Figure 1(b)) expression were both significantly downregulated in colorectal cancer tissues. Particularly, the expression levels of miR-15a in colorectal cancer tissues were positively correlated with those of miR-16 significantly (Spearman correlation coefficient *r* = 0.652, *P* < 0.001, Figure 1(c)).

Then, we chose the median values of miR-15a (2.39) and miR-16 (1.93) expression as the cutoff points. Colorectal cancer patients with an expression level exceeding the median values for miR-15a or miR-16 were deemed to be high expressions of miR-15a or miR-16; all other patients were considered to be low expressions of miR-15a or miR-16. Of 126 patients with colorectal cancer, 53 (42.06%) were low expression of both miR-15a and miR-16 (miR-15a-low/miR-16-low), 30 (23.81%) were high expression of both miR-15a and miR-16 (miR-15a-high/miR-16-high), 23 (18.25%) were miR-15a-high and miR-16-low expression (miR-15a-high/miR-16-low), and 20 (15.87%) were miR-15a-low and miR-16-high expression (miR-15a-low/miR-16-high).

### 3.2. Associations of Combined miR-15a and miR-16 Expression with the Clinicopathological Features of Colorectal Cancer

The associations of miR-15a and/or miR-16 expression with the clinicopathological features of colorectal cancer were summarized in Table 1. Low miR-15a expression and low miR-16 expression were both significantly associated with advanced TNM stage (both *P* = 0.001, Table 1), poorly histological grade (both *P* = 0.02, Table 1), and the presence of lymph node metastasis (both *P* = 0.01, Table 1) of patients with colorectal cancer. More importantly, combined downregulation of miR-15a and miR-16 (miR-15a-low/miR-16-low) was found in 53 patients (42.06%), who more frequently had advanced TNM stage (*P* < 0.001, Table 1), poorly histological grade (*P* = 0.01, Table 1), and positive lymph node metastasis (*P* = 0.008, Table 1) than those with other expression patterns for these two miRNAs. However, there were no significant associations between patients' gender, age, site of primary tumor, tumor size or depth of tumor invasion, and the combined expression patterns of miR-15a and miR-16.

### 3.3. Prognostic Implications of Combined miR-15a and miR-16 Expression in Colorectal Cancer

**Figure 2:**
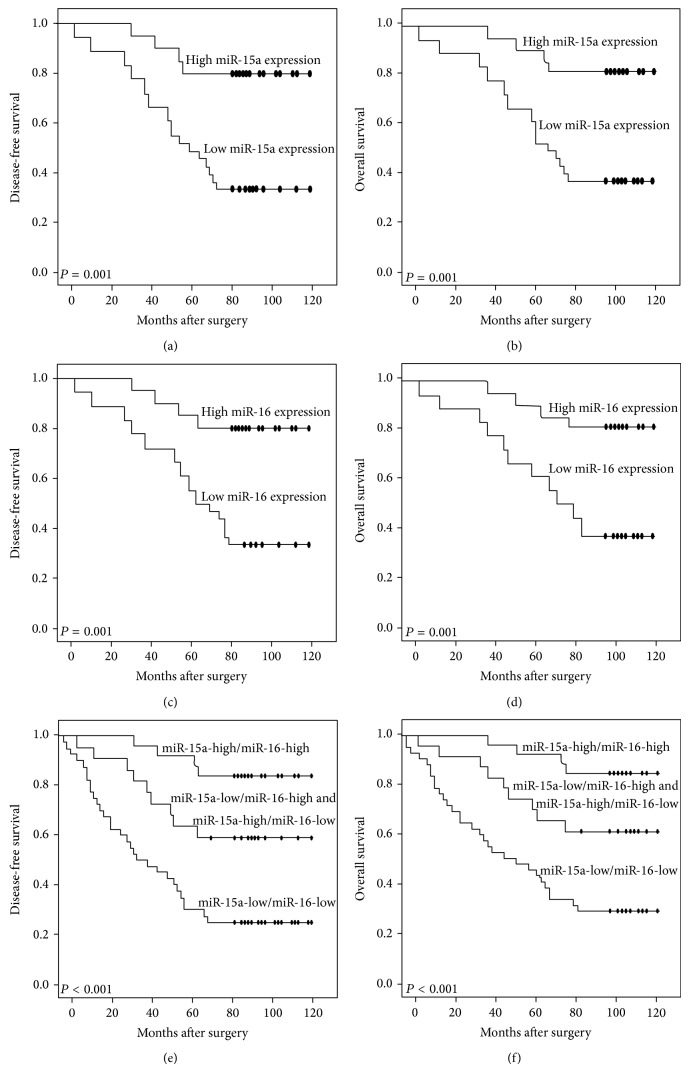
Kaplan-Meier survival curves of the disease-free survival and overall survival for miR-15a expression ((a) and (b), resp.), miR-16 expression ((c) and (d), resp.), and combined expression miR-15a and miR-16 (miR-15a/miR-16 (e) and (f), resp.) in colorectal cancer.

The potential prognostic impacts of miR-15a and/or miR-16 expression in colorectal cancer were evaluated by the Kaplan-Meier method. As a result, DFS and OS of patients with low miR-15a expression (both *P* = 0.001, Figures 2(a) and 2(b), resp.) or low miR-16 expression (both *P* = 0.001, Figures 2(c) and 2(d), resp.) were both significantly shorter than those of patients with either high miR-15a expression or high miR-16 expression. More importantly, patients with combined miR-15a-low/miR-16-low expression had the worst DFS and OS compared to other two groups (miR-15a-high/miR-16-high, miR-15a-low(high)/miR-16-high(low); both *P* < 0.001; Figures 2(e) and 2(f), resp.).

Moreover, univariate analysis showed that lymph node metastasis (*P* = 0.01 and 0.008, resp.), TNM stage (both *P* < 0.001), histological grade (*P* = 0.006 and 0.001, resp.), low miR-15a expression (both *P* = 0.001), low miR-16 expression (both *P* = 0.001), and combined miR-15a-low/miR-16-low expression (both *P* < 0.001) were all factors with significant impacts on DFS and OS (Table 2). When multivariate analysis was done according to the Cox hazard model, the TNM stage was excluded because it incorporates the clinicopathological factor of lymph node metastasis. As shown in Table 2, lymph node metastasis (*P* = 0.02 and 0.01, resp.), histological grade (both *P* = 0.01), miR-15a expression (both *P* = 0.01), miR-16 expression (both *P* = 0.01), and combined miR-15a/miR-16 expression (both *P* < 0.001) were all independent prognostic factors for both DFS and OS in patients with colorectal cancer. Notably, the prognostic impact of combined miR-15a/miR-16 expression for patients with colorectal cancer was significantly higher than that of miR-15a expression or miR-16 expression alone.

## 4. Discussion

Growing evidence has demonstrated that tumorigenesis and progression of human colorectal cancer are affected by disturbances in the regulatory network of miRNAs and their target genes. However, there is little information available regarding miRNAs in this malignancy. In the present study, we evaluated the expression levels of miR-15a and miR-16 and then correlated their expression levels to the clinicopathological parameters of patients with colorectal cancer. After the retrospective study with 126 patients with colorectal cancer, a combined analysis of miR-15a and miR-16 expression emerged as a significantly predictive panel for prognosis in colorectal cancer. It also served as an independent prognostic factor of colorectal cancer besides lymph node metastasis and histological grade. To the best of our knowledge, this is the first study showing the clinical value of miR-15a and miR-16 expression in a large series of patients with colorectal cancer.

It is well known that the importance of miRNAs in cancer, in general, is highlighted by the observation of the altered expression patterns of miRNAs in malignant cells. miR-15a and miR-16, transcribed as a cluster (miR-15a/miR-16), was originally identified as potential cancer genes in the pathogenesis of chronic lymphocytic leukemia [11], which provided the first evidence that these two miRNAs might be important for tumorigenesis. After that, accumulating studies reported the aberrant expression of miR-15a and miR-16 in various human cancers and the functional relevance of such aberrations [11–16]. For example, Bandi and Vassella showed that miR-15a/miR-16 were frequently deleted or downregulated in squamous cell carcinomas and adenocarcinomas of the lung. They also confirmed that G1 cyclins were major targets of miR-15a/miR-16 in non-small cell lung cancer suggesting that this cluster might be implicated in cell cycle control and contribute to the tumorigenesis of this malignancy [14]. Bhattacharya and colleagues observed the reduced expression of miR-15a and miR-16 in ovarian cell lines and in primary ovarian tissues and found that these miRNAs could inhibit ovarian cancer cell proliferation and clonal growth by targeting Bmi-1 [21]. Sun and colleagues reported that miR-15a and miR-16 could affect the angiogenesis of multiple myeloma by targeting VEGF [22]. Musumeci and colleagues suggested a molecular circuitry in which miR-15a and miR-16 and their correlated targets cooperate to promote tumor expansion and invasiveness of prostate cancer through the concurrent activity on stromal and cancer cells [16]. Bonci and colleagues also proposed that miR-15a and miR-16 might act as tumor suppressor genes in prostate cancer through the control of cell survival, proliferation, and invasion [23]. These findings suggest that miR-15a and miR-16 may have cell type-specific functions.

Although much is known about the aberrant expression of miR-15a and miR-16 in human colorectal cancer, much less is known about the clinical relevance of such aberrations. In the current study, our data, based on a large cohort of patients with colorectal cancer, confirmed the downregulation of both miR-15a and miR-16 in malignant tissues compared with adjacent normal colorectal mucosa. We also found that miR-15a and/or miR-16 downregulation were all associated with positive lymph node metastasis, advanced TNM stage, and poorly histological grade, which are all parameters representing tumor aggressiveness. The findings suggested that both miR-15a and miR-16 are implicated in tumorigenesis and tumor progression of colorectal cancer. The same chromosome region of the two miRNAs allowed us to hypothesize that the miR-15a/miR-16 cluster could play an important role in colorectal cancer. Moreover, the survival analysis showed that miR-15a and/or miR-16 downregulation all could predict shorter DFS and OS than the corresponding controls. The Cox proportional hazard model further demonstrated that colorectal cancer patients with miR-15a-low/miR-16-low expression had higher relative risk of death than other expression patterns. More importantly, the prognostic value of combined miR-15a/miR-16 expression in this cancer was more significant than that of miR-15a or miR-16 expression alone, implying that a combined analysis of miR-15a and miR-16 expression status may enhance our accuracy in identifying patients at high risk of aggressive tumor progression and poor prognosis and hence provide useful information for clinical management.

In conclusion, our results offer the convincing evidence that the aberrant expression of miR-15a and miR-16 could be used to stratify patients with aggressive tumor progression of colorectal cancer. The combined pattern of miR-15a and miR-16 downregulation has a significant value for distinguishing patients with a worse prognosis of colorectal cancer after surgery. Further investigation on the molecular mechanisms by which miR-15a/miR-16 are implicated in colorectal carcinogenesis is required.

## Figures and Tables

**Table 1:** Associations of miR-15a and/or miR-16 expression with the clinicopathological characteristics of colorectal cancer.

Clinical features	Case number (%)	miR-15a-low		miR-16-low		miR-15a-low/miR-16-low	
		(*N*, %)	*P*	(*N*, %)	*P*	(*N*, %)	*P*
Gender							
Male	76 (60.32)	42 (55.26)	NS	45 (59.21)	NS	32 (42.11)	NS
Female	50 (39.68)	31 (62.00)		31 (62.00)		21 (42.00)	
Age (years)							
<66	48 (30.10)	32 (66.67)	NS	32 (66.67)	NS	22 (45.83)	NS
≥66	78 (69.90)	41 (52.56)		44 (56.41)		31 (39.74)	
Site of primary tumor							
Right-sided	48 (38.10)	30 (62.50)	NS	30 (62.50)	NS	22 (45.83)	NS
Left-sided	46 (36.51)	26 (56.52)		26 (56.52)		20 (43.48)	
Rectum	32 (25.40)	17 (53.13)		20 (62.50)		11 (34.38)	
Tumor size (cm)							
<5	70 (55.56)	42 (60.00)	NS	42 (60.00)	NS	32 (45.71)	NS
≥5	56 (44.44)	31 (55.36)		34 (60.71)		21 (37.50)	
Depth of tumor invasion							
T1-T2	47 (37.30)	30 (63.83)	NS	30 (63.83)	NS	20 (42.55)	NS
T3-T4	79 (62.70)	43 (54.43)		46 (58.23)		33 (41.77)	
Lymph node metastasis							
Negative	68 (53.97)	29 (42.65)	0.01	29 (42.65)	0.01	13 (19.12)	0.008
Positive	58 (46.03)	44 (75.86)		47 (81.03)		40 (68.97)	
TNM stage							
I	13 (10.32)	3 (23.08)	0.001	3 (23.08)	0.001	0 (0)	<0.001
II	46 (36.51)	17 (36.96)		20 (43.48)		10 (18.87)	
III	50 (39.68)	36 (72.00)		36 (72.00)		26 (52.00)	
IV	17 (13.49)	17 (100.00)		17 (100.00)		17 (100.00)	
Histological grade							
Well/moderately	80 (63.49)	40 (50.00)	0.02	40 (50.00)	0.02	23 (28.75)	0.01
Poorly	46 (36.51)	33 (71.74)		36 (78.26)		30 (65.22)	

Note: “NS” refers to the difference without statistical significance.

**Table 2:** Prognostic value of combined miR-15a and miR-16 expression for the disease-free survival and overall survival in univariate and multivariate analyses by Cox regression.

	Disease-free survival		Overall survival	
	Hazard ratio (95% CI)	*P*	Hazard ratio (95% CI)	*P*
Univariate				
Gender	1.005 (0.101–2.208)	NS	1.298 (0.122–2.891)	NS
Age	1.091 (0.198–2.623)	NS	1.255 (0.226–2.879)	NS
Site of primary tumor	0.698 (0.119–5.516)	NS	0.869 (0.126–1.881)	NS
Tumor size	0.379 (0.103–0.743)	NS	0.799 (0.136–1.606)	NS
Depth of tumor invasion	0.462 (0.102–1.039)	NS	0.729 (0.108–1.692)	NS
Lymph node metastasis	3.232 (1.566–6.676)	0.01	3.528 (1.616–7.869)	0.008
TNM stage	5.069 (1.981–12.620)	<0.001	5.869 (1.996–12.788)	<0.001
Histological grade	3.982 (1.119–8.116)	0.006	4.569 (1.126–10.281)	0.001
miR-15a	4.336 (1.201–9.682)	0.001	4.628 (1.128–10.161)	0.001
miR-16	4.283 (1.123–9.416)	0.001	4.562 (1.121–10.286)	0.001
miR-15a/miR-16	5.019 (1.972–12.069)	<0.001	5.712 (1.982–12.689)	<0.001
Multivariate				
Lymph node metastasis	2.058 (1.013–4.621)	0.02	2.655 (1.039–5.528)	0.01
Histological grade	2.698 (1.119–5.516)	0.01	2.869 (1.126–5.881)	0.01
miR-15a	2.782 (1.122–6.031)	0.01	3.016 (1.129–6.616)	0.01
miR-16	2.598 (1.013–6.006)	0.01	2.912 (1.110–6.011)	0.01
miR-15a/miR-16	4.818 (1.937–10.129)	<0.001	4.983 (1.946–10.962)	<0.001
